# Successful Control of External Biliary Fistula by Using SMS 201-995 in a Child

**DOI:** 10.1155/1996/80945

**Published:** 1996

**Authors:** Mohamad Abid Bakhotmah

**Affiliations:** King Abdulaziz University Hospital Department of Surgery P.O. Box 6615 Jeddah 21452 Saudi Arabia

## Abstract

Somatostatin is a potent inhibitory hormone, it's synthetic analogue is more potent and has a prolonged action. It has a wide range of actions in the gastrointestinal system; among which is an anticholeritic action on bile secretion.

The use of Sandostatin in the management of a case of complicated biliary fistula is reported; it controlled the fistula reducing its daily output from 200 ml to less than 5 ml per day.


*HPB Surgery*, 1996, Vol.9, pp.183–184
Reprints available directly from the publisher
Photocopying permitted by license only

© 1996 (OPA (Overseas Publishers Association)
Amsterdam B.V. Published in The Netherlands
by Harwood Academic Publishers GmbH
Printed in Malaysia

### CASE REPORT

### Successful Control of External Biliary Fistula by Using SMS 201–995 in a Child

MOHAMAD ABID BAKHOTMAH

Consultant Hepatobiliary Surgeon and Assistant Professor

*(Received 23 October 1993)*

Somatostatin is a potent inhibitory hormone, it's synthetic analogue is more potent and has a prolonged action. It has a wide range of actions in the gastrointestinal system; among which is an anticholeritic action on bile secretion.

The use of Sandostatin in the management of a case of complicated biliary fistula is reported; it controlled the fistula reducing its daily output from 200 ml to less than 5 ml per day.

KEY WORDS: Cholelithiasis common bile duct calculi sphincterotomy endo scopic cholangiopancreaticography endoscopic retrograde

### CASE REPORT

A 4-year old boy was referred to our hospital with abdominal distention and failure to thrive since he was 3 years old. Liver biopsy was done at the referring hospital which was in conclusive. On examination, there was a cystic mass occupying the Right side of the abdomen causing the distention, there was no ascites; ultrasonography, CT scan, HIDA scan suggested the diagnosis of Caroli's disease because of the bilateral intrahepatic saccular dilatation of the biliary tree. The abdominal CT and ultrasonography demonstrated the presence of a large cystic mass in the sub-hepatic region extending to the pelvic inlet but its origin could not be identified.

At laparotomy three communicating cystic masses were found in the Right side of abdomen inferior to the liver and full of bile, but not communicating with the extra-hepatic biliary tree which looked quite normal. The cysts were excised after dissecting them from the inferior surface of the right lobe. The dissection was not difficult. A definitive communication between the liver surface and the cystic mass could not be identified during surgery but of course, this does not exclude it's presence.

A small butterfly needle was inserted into the CBD to perform cholangiography, which showed 3 filling defects. The stones were removed through a choledochotomy and a T-tube was inserted. The gallbladder was not removed and no biliary enteric anastomosis performed. Postoperatively, the histology report of the excised cysts was that it was typical of Caroli's disease.

The T-tube cholangiography showed no evidence of stones in the CBD and contrast was seen in the duodenum. When, the T-tube was removed the tract persisted as a biliary fistula which was resistant to any attempt to close it including cholecystojujenostomy; done as a 2nd operation, the output was 150–200 mls. per day and that was for almost 12 months. During the late course of his illness, the child developed generalized psoriatic skin-lesions which was then diagnosed as due to histocystosis-X. Bone marrow examination confirmed that the disease had spread to bone marrow. Treatment for histocystosis-X by cytotoxic therapy was given but it had no effect on the fistula.

*Correspondence to*: Mohamad A. Bakhotmah, King Abdulaziz University Hospital, Department of Surgery, P.O. Box 6615, Jeddah 21452, Saudi Arabia

The child started on Sandostatin 10 μgm 6 hourly subcutaneously. Within less than 48 hours, the fistula output markedly reduced to less than 5 mls. per day. No collection bag was needed only a gauze dressing. The patient was on Sandostatin for more than one year before he died due to extensive spread of histocystosis-X.

### DISCUSSION

Sandostatin (SMS 201–995) is the synthetic analogue of the naturally occurring peptide Somatostatin. It has the same action as Somatostatin but is more potent and has a prolonged action.

It's effect on bile secretion has been studied in several experimental models, it reduces bile secretion in rats[1] and has an anticholeretic effect in dogs[2].

In man, Somatostatin reduces the bile secretion by 30–40%, it acts on the bile acid-dependent canalicular bile secretion and also on the bile acid-independent ductular secretion[3] SMS-201–995 almost abolishes the cholecystokinin release and gallbladder contraction[4].

Inspite of such evidences in animal and normal humans, there is only one case report of using Sandostatin in biliary fistula[5].

This case is the second and for the first time, it is used for a long time in a paediatric patient with biliary fistula.

Although it did not heal the fistula; there are advantages for using Sandostatin; the metabolic and skin complications of high output biliary fistula are avoided, the patient can eat normally and there is no need for prolonged TPN, there is no need for a collection bag or PTC to collect the bile as the amount is minimal and daily dressing is sufficient; and because the drug is given subcutaneously hospitalization is also not needed. The non-healing may be due to infiltration of the tract by histocystosis-X or due to inadequate distal drainage. The biliary enteric anastomosis-choledochoduodenostomy or choledochojujenustomy–was not performed in the 1st operation because of fear of causing recurrent cholangitis[6] in a young child, of the second attempt, it was very difficult to reach the common bile duct so cholecystojujenostomy was performed but it also failed to control the fistula.

At the beginning of the treatment with Sandostatin, the patient had abdominal pain which is controlled by "Buscopan" suppositories given prior to the Sandostatin dose. This was needed only in the first 2 weeks, no side effect were seen after prolonged use of this a high dose, not even gallstones.

The use of Sandostatin is to be recommended in cases of biliary fistula and there is no need to deprive the patient of oral intake, although healing has not been accomplished in this case, control is adequate and healing may be accomplished in other cases.

### REFERENCES

1. Schirmer B.D, Kortz W.J, Miller R, *et al.* (1985) Is Somatostatin a directly acting cholestatic hormone? *J Clin Res,* **39**: 237–245.
2. Holm I, Thulin Z, Sammengard H. (1978) Anticholeretic effect of Somatostatin in anesthetized dogs. *Acta phisiol Scand,* **104:** 241–243.
3. Nyberg B. (1990) Bile secretion in man, the effects of Somatostatin, vasoactive intestinal peptide and secretion. *Acta Chair Scand supp,* 557 1–40.
4. Lembacke B, Creutzfeldt W, Scleser S. *et al.* (1987) The effect of the long acting somatostatin analogue Sandostatin (SMS 201–995). Digestion, **36** (2) 108–24.
5. Miranda-Rutz, Castanon-Gonzales J, Pereze C, *et al.* (1990) Effect of a prolonged-action synthetic Somatostatin analogue (SMS 201–995) on biliary output in a patient with external bile duct fistula. *Rev Gastroenterol Mex Apr-Jun,* 55 (2) 67–9.
6. Mercadier M, Chigoz J.P, Clot M.D. *et al.* (1984)*Carolie's Disease: World Journal of Surgery.* **8** (1):22–29.

